# Synergistic Antitumour Properties of *viscumTT* in Alveolar Rhabdomyosarcoma

**DOI:** 10.1155/2017/4874280

**Published:** 2017-07-16

**Authors:** Rahel Mascha Stammer, Susann Kleinsimon, Jana Rolff, Sebastian Jäger, Angelika Eggert, Georg Seifert, Catharina I. Delebinski

**Affiliations:** ^1^Department of Paediatric Oncology/Haematology, Otto-Heubner Centre for Paediatric and Adolescent Medicine (OHC), Charité-Universitätsmedizin Berlin, Augustenburger Platz 1, 13353 Berlin, Germany; ^2^EPO GmbH, Experimental Pharmacology & Oncology, Robert-Rössle-Str. 10, 13125 Berlin-Buch, Germany; ^3^Birken AG, Streiflingsweg 11, 75223 Niefern-Öschelbronn, Germany

## Abstract

Aqueous mistletoe extracts from the European mistletoe (*Viscum album*) contain mainly mistletoe lectins and viscotoxins as cytotoxic compounds. Lipophilic triterpene acids, which do not occur in conventional mistletoe preparations, were solubilised with *β*-cyclodextrins. The combination of an aqueous extract (*viscum*) and a triterpene-containing extract (*TT*) recreated a whole mistletoe extract (*viscumTT*). These extracts were tested on rhabdomyosarcoma in vitro, ex vivo, and in vivo with regard to anticancer effects. *Viscum* and *viscumTT* inhibited cell proliferation and induced apoptosis effectively in a dose-dependent manner in vitro and ex vivo, whereas *TT* showed only moderate inhibitory effects. *viscumTT* proved to be more effective than the single extracts and displayed a synergistic effect in vitro and a stronger effect in vivo. *viscumTT* induced apoptosis via the extrinsic and intrinsic pathways, evidenced by the loss of mitochondrial membrane potential and activation of CASP8 and CASP9. CASP10 inhibitor inhibited apoptosis effectively, emphasising the importance of CASP10 in *viscumTT*-induced apoptosis. Additionally, *viscumTT* changed the ratio of apoptosis-associated proteins by downregulation of antiapoptotic proteins such as XIAP and BIRC5, thus shifting the balance towards apoptosis. *viscumTT* effectively reduced tumour volume in patient-derived xenografts in vivo and may be considered a promising substance for rhabdomyosarcoma therapy.

## 1. Introduction

Rhabdomyosarcoma (RMS) is the most common soft tissue sarcoma in children and adolescents in Germany, accounting for 3.1% of all paediatric tumours [1]. Alveolar RMS (ARMS) is one of the two main histopathological subtypes and occurs in approximately 25% of the cases [2–4]. ARMS is more common in adolescents and young adults and associated with aggressive clinical behaviour and poor treatment response and outcome [5]. Most of ARMS are characterised by the balanced translocations t(2;13)(q35;q14) or t(1;13)(q36;14) [6], resulting in the PAX3/FOXO1 (~55%) or PAX7/FOXO1 (~25%) fusion genes, respectively [7]. Despite aggressive multimodal treatment regimes, the prognosis of fusion-positive ARMS (65%) [8] and patients with metastatic disease (<20%) [9] remains poor and has not changed in years [10], highlighting the need for new therapy approaches.

Aqueous extracts of the European mistletoe (*Viscum album*) are widely used in complementary cancer therapy in German-speaking countries, despite their controversial efficacy since 1920. *Viscum album* contains a large variety of biological active compounds including mistletoe lectins (ML) [11], viscotoxins (VT) [12–14], triterpene acids [15, 16], flavonoids [17, 18], and polysaccharides [19, 20]. Commercially available mistletoe preparations are based on aqueous extracts and contain mainly ML and VT as active compounds. Mistletoe extracts have been shown to exert cytotoxic effects on acute leukaemia [21], multiple myeloma [22], head and neck squamous cell carcinomas [23], and bladder carcinoma [24] in vitro as well as acute leukaemia [25], breast cancer [26], malignant melanoma [27], and pancreatic carcinoma [28] in vivo. Furthermore, there is clinical evidence that *Viscum album* extracts prolong the overall survival of patients with pancreatic cancer [29].

Lipophilic triterpene acids, such as oleanolic acid (OA) and betulinic acid (BA), represent another group of mistletoe-derived substances with known cytotoxic effects. Previous studies on neuroblastoma [30, 31], osteosarcoma [32], acute leukaemia [33], breast cancer [34], and gastric cancer [35] showed that OA and its derivatives inhibit cell proliferation and induce apoptosis effectively in vitro. Moreover, in vivo experiments have shown cytotoxic effects of OA on osteosarcoma [36], gallbladder carcinoma [37], hepatocellular carcinoma [38], and pancreatic carcinoma [39]. Due to their low solubility, triterpene acids do not occur in significant amounts in commercial mistletoe extracts [40]. Using cyclodextrins, it was possible to solubilise triterpene acids and therefore to overcome their loss in standardised aqueous extracts.

Previous studies showed the efficacy of a combined mistletoe extract produced by addition of solubilised triterpene acids (*TT*) to aqueous mistletoe extracts (*viscum*), creating a whole plant extract (*viscumTT*). This whole plant extract effectively induced apoptosis in acute myeloid and lymphoblastic leukaemia [41, 42], Ewing-sarcoma [43], and osteosarcoma [44] and inhibited tumour growth in murine melanoma in vivo [45]. The aim of this study was to analyse the effect of the whole mistletoe extract, *viscumTT*, on rhabdomyosarcoma in vitro, ex vivo, and in vivo.

## 2. Materials and Methods

### 2.1. Material and Reagents

RPMI 1640, penicillin, streptomycin, trypsin (0.05%), and phosphate-buffered saline (PBS) were purchased from Gibco®, Life Technologies (Darmstadt, Germany). Foetal calf serum (FCS) was obtained from Biochrom (Berlin, Germany). RIPA buffer, protease inhibitors, molecular mass standards for SDS-PAGE, sodium dodecyl sulfate (SDS), dimethyl sulfoxide (DMSO), 5,6,6-tetrachloro-1,1,3,3-tetraethylbenzimidazolcarbocyanine iodide (JC-1), carbonyl cyanide 3-chlorophenylhydrazone (CCCP), and propidium iodide (PI) were purchased from Sigma-Aldrich (München, Germany). Tween, acrylamide, and dithiotreitol (DTT) were purchased from Carl Roth GmbH (Karlsruhe, Germany).

### 2.2. *Viscum album* Extracts

*Viscum album* extracts were kindly provided by Birken AG (Niefern-Öschelbronn, Germany) and prepared from *Viscum album* harvested from apple trees (*Malus domestica* Borkh.) as previously described [41, 42, 46]. Intact mistletoe lectin-I (A + B chain) was quantified by ELISA in the *viscum* extract as described before [47]. OA and BA were quantified in the *TT* extract by gas chromatography-flame ionisation detector (GC-FID) and external calibration with OA as reference substance [40]. Both extracts, *viscum* and *TT*, were reconstituted in PBS to a final concentration of 1 *μ*g/mL intact ML-I and <1 *μ*g/mL VT in *viscum* and 4000 *μ*g/mL OA and 172 *μ*g/mL BA in *TT* (Table 1).

### 2.3. Cell Culture

Two human ARMS cell lines were used in this study. RH-30 cells were obtained from the German Collection of Microorganisms and Cell Cultures (DSMZ, Braunschweig, Germany) and RMS-13 cells from the American Type Culture Collection (ATCC, Manassas, VA, USA). Both cell lines were maintained in RPMI 1640 medium with L-glutamine, supplemented with 10% heat-inactivated FCS, 100 U/mL penicillin, and 100 *μ*g/mL streptomycin. For experimental settings, 2 × 10^5^/mL cells were seeded onto 6-well plates with a final volume of 2 mL/well. Cell numbers were adjusted to sizes of well plates according to experimental settings. Cells were cultured 24 h to allow cell attachment, then *viscum*, *TT*, and *viscumTT* were added in defined concentrations for another 24 h.

### 2.4. Determination of Cell Proliferation and Measurement of Early Cytotoxicity

Cell counts were determined with the CASY® Cell Counter and Analyser System of Schärfe System GmbH (Reutlingen, Germany). Alterations of cell proliferation were indicated as the percentage of untreated control cells (100% viability). To rule out early cytotoxicity, lactate dehydrogenase (LDH) release was measured after 2 h of incubation with *viscum*, *TT*, and *viscumTT* using the Cytotoxicity Detection Kit (Roche Diagnostics GmbH, Mannheim, Germany) according to the manufacturer's instructions.

### 2.5. Determination of Apoptosis

After 24 h incubation with *viscum*, *TT*, and *viscumTT* in increasing concentrations, cells were stained with APC-conjugated annexin V (BD Biosciences, Heidelberg, Germany) and 1 mg/mL PI as described before [42]. Cells were analysed by flow cytometry (FACSCalibur™, Becton Dickinson, Heidelberg, Germany), and results were evaluated with FlowJo® Software (Tree Star, Inc., Ashland, USA).

### 2.6. Assessment of Mitochondrial Membrane Potential (∆*Ψ*_m_)

Changes in mitochondrial membrane potential (∆*Ψ*_m_) were detected by JC-1 staining and flow cytometry, according to the manufacturer's instructions. Subsequently, results were evaluated with FlowJo Software. 50 *μ*M depolarising CCCP was used as a reference control.

### 2.7. Determination of Caspase Activity

Activity of caspase-3 (CASP3), caspase-8 (CASP8), and caspase-9 (CASP9) was measured using the Green Caspase Staining Kit (Promokine, Heidelberg, Germany) according to the manufacturer's protocol. Subsequently, cells were analysed by flow cytometry and results were evaluated with FlowJo Software.

### 2.8. Caspase Inhibitor Assays

Cells were pretreated for one hour with 50–100 *μ*M caspase inhibitors, before 24 h incubation with *viscum* (2.5–5 ng/mL) and *viscumTT* (2.5–5 ng/mL *viscum* + 35–40 *μ*g/mL *TT*). DMSO was added as a solvent control. Induction of apoptosis was determined by annexin V/PI staining and flow cytometry analysis as described above. In this experimental setting, we used, in addition to a pan-caspase inhibitor (Z-VAD-FMK), inhibitors of CASP8 (Z-IETD-FMK), CASP9 (Z-LEHD-FMK), and caspase-10 (CASP10; Z-AEVD-FMK) (R&D Systems, Minneapolis, MN, USA).

### 2.9. Protein Extraction and Western Blot Analyses

Cells were incubated with *TT* (OA 40 *μ*g/mL), *viscum* (ML 5 ng/mL), and *viscumTT* (OA 40 *μ*g/mL + ML 5 ng/mL) for 24 h. After washing twice with PBS, cells were lysed for 30 min in RIPA buffer containing protease inhibitors. Protein lysates were centrifuged at 14000 *rpm* for 5 min at 4°C, then protein concentrations were determined by Bradford Reagent (Bio-Rad, München, Germany). Lysates (20–25 *μ*g protein/lane) were separated by SDS-PAGE and transferred to nitrocellulose membranes (Bio-Rad). Blots were incubated with primary antibodies overnight at 4°C. After incubation with HRP-conjugated secondary antibodies (Bio-Rad), protein bands were visualized by ECL (Thermo Fisher Scientific, Bonn, Germany) and Molecular Imager® ChemiDoc™ (Bio-Rad). The following primary antibodies were used: *β*-actin (#A3854, Sigma-Aldrich), B-cell lymphoma 2 (BCL2; #2870, Cell Signaling Technology, CST, Danvers, MA, USA), BCL2 like 1 (BCL2L1; #2764, CST), baculoviral IAP repeat containing 5 (BIRC5; #2803, CST), myeloid cell leukemia 1 (MCL1; sc-819, Santa Cruz Biotechnology, Santa Cruz, CA, USA), poly(ADP-ribose) polymerase 1 (PARP1; #9542, CST), and X-linked inhibitor of apoptosis protein (XIAP; #610716, BD Biosciences).

### 2.10. Ex Vivo Cultured RMS Primary Cells

Tumour samples were obtained as a treatment residue from three different patients with ARMS during routine surgical resection. Tumour tissue was not explicitly collected for this study, and diagnoses were confirmed by histopathology by the institute of pathology, Charité - Universitätsmedizin Berlin. Immediately after surgical excision, tumour tissue from patient no. 1 (11 years, female, recurrent disease), patient no. 2 (12 years, male, primary disease), and patient no. 3 (13 years, female, primary disease) was cut into smaller pieces and cultured as primary explants in RPMI 1640 base medium with L-glutamine supplemented with 20% heat-inactivated FCS and 1% penicillin/streptomycin solution. When cells dissociated from the explant and formed a confluent monolayer culture, apoptosis induction was investigated. Ex vivo cell cultures were used for experimental settings within seven trypsinised passages. Because of slow or bad cell growth, experiments could only be performed twice. For analyses, cells were seeded into 12-well microtiter plates at 1.5 × 10^5^–3 × 10^5^ cells/well and incubated with increasing concentrations of *viscum*, *TT*, and *viscumTT* as described above. Subsequently, apoptosis was measured by annexin V/PI assay. Further, JC-1 measurement was carried out with cells from patient no. 1. In accordance with the Declaration of Helsinki, written informed consent was obtained from the patients and experiments were approved by the local ethics committee of the Charité - Universitätsmedizin Berlin.

### 2.11. RMS Xenografts and Experimental Procedures

Patient-derived RMS tissue was subcutaneously transplanted into the left flank of eight-week-old NMRI-nu/nu mice. The mice were obtained from Charles River Laboratories (Sulzfeld, Germany), housed in a pathogen-free facility under pathogen-free conditions, and fed autoclaved standard diet (Sniff, Soest, Germany) with acidified drinking water ad libitum. The tumour was propagated in vivo, and tumour tissue from one in vivo passage was used for s.c. implantation in the inguinal region of seven (no. 1) or five (no. 2) mice per treatment or control group. The same patient material was used for ex vivo as well as in vivo experiments (no. 1 ex vivo = no. 1 in vivo, no. 2 ex vivo = no. 2 in vivo). Treatment with the test substances or controls started on day 12 when tumours were palpable. Mice were treated with *viscum*, *TT*, *viscumTT*, and cyclodextrins (control group) intratumourally (i.t) and doxorubicin (Doxo) intravenously (i.v.) in no. 1. In experiment no. 2, mice received *viscumTT* i.t. and *viscumTT* and cyclodextrins i.v. The mice were treated every two to three days in rising concentrations, and each dose was given twice. The administered concentrations were 40/60/80 mg/kg OA (*TT*), 0.5/1.0/1.5 *μ*g/kg ML (*viscum*), or a combination thereof (*viscumTT*). Body weight was measured before each treatment, and mice were carefully monitored for health and symptoms of toxicity. The animals were sacrificed by cervical dislocation at the end of the experiment or if the mice were moribund (tumour volume > 1.2 cm^3^ or >10% body weight loss). The animal experiments were carried out in accordance with the German legislation on the care and use of laboratory animals and in accordance with the United Kingdom Coordinating Committee on Cancer Research Guidelines for the Welfare of animals in Experimental Neoplasia to minimise suffering. Approval for the study was obtained from the Regional Office for Health and Social Affairs (LaGeSo, approval G-0030/15).

### 2.12. Statistics

All in vitro experiments were performed in independent experiments at least three times, for which means ± standard error are displayed in bar graphs. Ex vivo experiments were carried out only in sets of two independent experiments due to low cell growth. To assess whether the effect of *viscumTT* was synergistic, Webb's fractional product (^∗^Fp) [48] was calculated as described before [42]. Values greater than one (^∗^Fp > 1) display a synergistic effect. Two-way ANOVA and Bonferroni post hoc tests were applied to determine differences between mouse xenograft treatment groups. All results with *p* ≤ 0.05 were considered significant.

## 3. Results

### 3.1. *viscumTT* Inhibits Cell Proliferation and Induces Apoptosis Synergistically In Vitro

**Figure 1:**
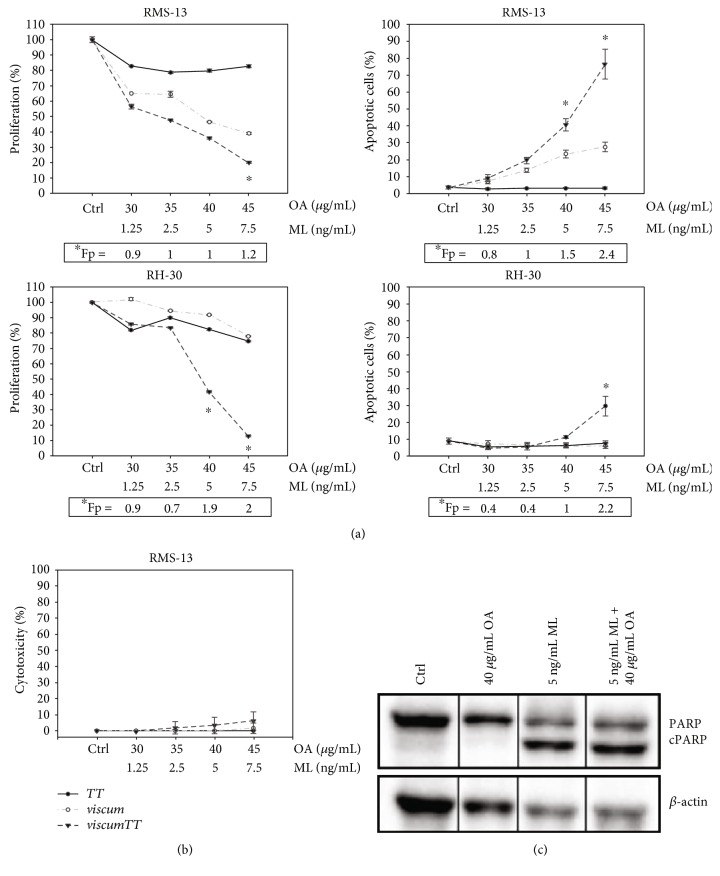
*viscumTT* inhibits cell proliferation and induces apoptosis synergistically in ARMS. (a) RMS-13 and RH-30 cells were incubated with *viscum*, *TT*, and *viscumTT* in increasing concentrations for 24 h. Cell proliferation was measured by CASY cell counter analysis, and the proliferation rate was calculated from the total cell numbers compared to untreated control cells. Apoptosis was assessed by annexin V/PI assay, and results were analysed by FlowJo Software. (b) RMS-13 cells were treated with *viscum*, *TT*, and *viscumTT* in increasing concentrations, and early cytotoxicity was measured by LDH assay. Results are presented as percentage of untreated control cells. (c) Apoptosis was confirmed by PARP cleavage. For this purpose, Western blot analyses were performed after treatment of RMS-13 cells with *viscum*, *TT*, and *viscumTT*. *β*-actin served as a loading control. Results are presented as means ± SD of three independent experiments. Webb's fractional product (Fp) was calculated to assess synergism, values ^∗^Fp > 1 display synergism. Mistletoe lectin (ML) and oleanolic acid (OA) concentrations were used as marker substances for *viscum* and *TT*, respectively.

To assess the antiproliferative effects of mistletoe extracts, RMS-13 and RH-30 cells were incubated with increasing concentrations of *viscum*, *TT*, and *viscumTT*. Both, *viscum* and *viscumTT*, effectively inhibited proliferation in a dose-dependent manner in RMS-13 cells, whereas *TT* showed only moderate inhibitory effects (Figure 1(a)). RH-30 cells were more resistant towards treatment with *viscum* or *TT*, while *viscumTT* showed a strong inhibitory effect. Notably, treatment with *viscumTT* led to a synergistic inhibitory effect on cell proliferation in both cell lines in higher concentrations (^∗^Fp > 1).

To determine the induction of apoptosis, RMS-13 and RH-30 cells were analysed by annexin V/PI staining and FACS analysis after 24 h of incubation with *viscum*, *TT*, and *viscumTT*. Neither *viscum* nor *TT* affected apoptosis in RH-30 cells; however, *viscumTT* induced apoptosis at high concentration ranges (Figure 1(a)). In comparison, RMS-13 cells reacted more sensitively to incubation with *viscum* and *viscumTT*, but treatment with *TT* also proved to be ineffective. *viscumTT* again displayed a synergistic effect on apoptosis induction in both cell lines at high concentration ranges. Detection of PARP cleavage by Western blot analysis confirmed apoptosis induction (Figure 1(c)).

To exclude an unwanted early cytotoxic effect via necrosis, LDH-release was measured after 2 h of incubation with *viscum*, *TT*, and *viscumTT* in RMS-13 cells. As shown in Figure 1(b), no significant LDH-release was detected; hence, a cytotoxic effect by necrosis could be excluded.

### 3.2. *viscumTT* Displays a Synergistic Effect on Mitochondrial Membrane Depolarisation and Caspase Activation In Vitro

**Figure 2:**
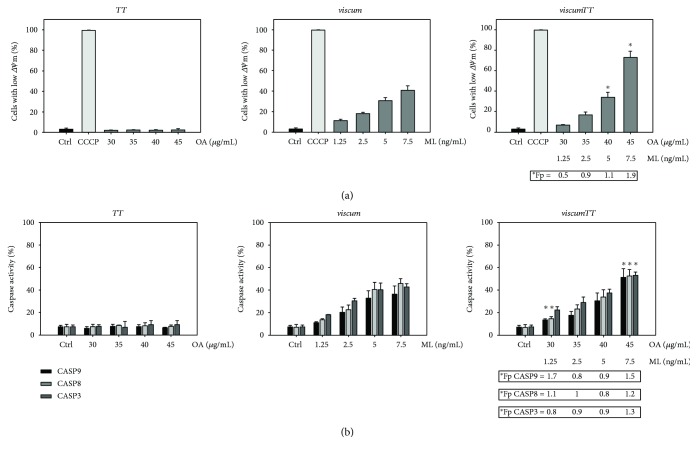
*viscumTT* displays synergistic effects on mitochondrial membrane (∆*Ψ*_m_) depolarisation and activation of CASP3, CASP8, and CASP9. RMS-13 cells were incubated with *viscum*, *TT*, and *viscumTT* in increasing concentrations for 24 h. (a) ∆*Ψ*_m_ depolarisation was analysed by JC-1 staining and flow cytometry. The protonophore carbonyl cyanide 3-chlorophenylhydrazone (CCCP), a mitochondrial membrane disruptor, was used as a positive control. (b) Active caspase assays were performed according to the manufacturer's instructions to monitor activation of caspase-3 (CASP3), caspase-8 (CASP8), and caspase-9 (CASP9). All results are presented as means ± SD of three independent experiments. Webb's fractional product (Fp) was calculated to assess synergism, values ^∗^Fp > 1 display synergism. Mistletoe lectin (ML) and oleanolic acid (OA) concentrations were used as marker substances for *viscum* and *TT*, respectively.

To evaluate the underlying mechanisms of *viscum-*, *TT-*, and *viscumTT*-induced cytotoxicity more closely, we analysed the involvement of mitochondria and caspases in apoptosis. For this purpose, RMS-13 cells were treated as described above and ∆*Ψ*_m_ loss was detected by JC-1 staining and FACS analysis. Treatment with *viscum*, and to an even more pronounced extent with *viscumTT*, led to a dose-dependent loss of ∆*Ψ*_m_, whereas *TT* showed no significant effect (Figure 2(a)).

Caspases play an essential role in apoptosis, since they are initiators (CASP9, CASP8) as well as effectors (CASP3). After 24 h treatment with *viscum*, *TT*, and *viscumTT*, caspase activation was assessed by active caspase assays and FACS analysis in RMS-13 cells. *Viscum* as well as *viscumTT* activated CASP3, CASP8, and CASP9 in a dose-dependent manner (Figure 2(b)). *viscumTT* displayed a synergistic effect on mitochondrial membrane depolarisation and caspase activation, confirming observations made in the previous experiments.

**Figure 3:**
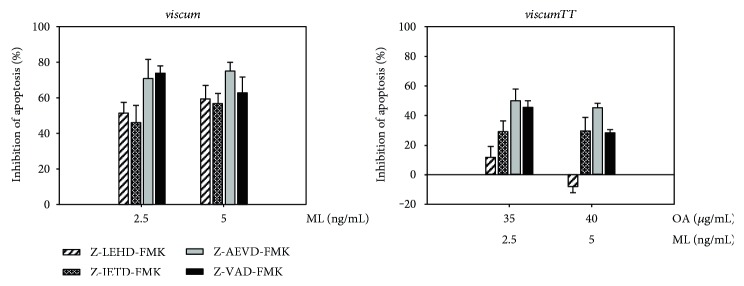
Caspase inhibitors effectively reduce *viscum*- and *viscumTT*-mediated apoptosis. RMS-13 cells were pretreated with 50–100 *μ*mol of caspase inhibitors Z-VAD-FMK (pan inhibitor), Z-IETD-FMK (caspase-8), Z-LEHD-FMK (caspase-9), or Z-AEVD-FMK (caspase-10) for one hour. Subsequently, cells were treated with *viscum*, *TT*, and *viscumTT* for 24 h and analysed by annexin/PI assay and flow cytometry. Results are presented as percentage of untreated control cells and means ± SD of three independent experiments. Mistletoe lectin (ML) and oleanolic acid (OA) concentrations were used as marker substances for *viscum* and *TT*, respectively.

To further determine caspase dependency in apoptosis induction, caspase inhibitor assays were performed and inhibition of apoptosis was estimated from untreated control cells. Interestingly, caspase inhibitors were more effective in preventing apoptosis in cells treated with *viscum* rather than *viscumTT*. The pan-caspase inhibitor achieved reduction of apoptosis by up to 73% in *viscum-* and 46% in *viscumTT*-treated cells (Figure 3). Inhibition of the extrinsic pathway by CASP10 and CASP8 inhibitors prevented apoptosis by up to 75% and 60%, respectively, after treatment with *viscum* and by 50% and 30%, respectively, in *viscumTT*-treated cells. Inhibition of the intrinsic apoptosis signalling pathway by CASP9 inhibitor was also effective in *viscum*-treated cells, but showed no effect after incubation with *viscumTT*. On the contrary, in increasing concentrations, CASP9 inhibitor enhanced *viscumTT*-induced apoptosis.

These results suggest that in addition to the intrinsic signalling pathway the extrinsic pathway plays a role in *viscum* and *viscumTT-*induced caspase-dependent apoptosis.

### 3.3. *Viscum album* Extracts Alter the Expression of Antiapoptotic Proteins

**Figure 4:**
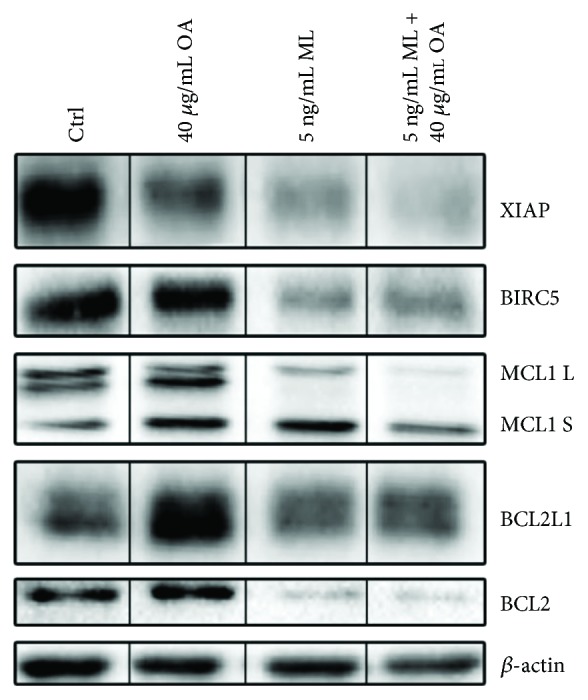
*viscumTT* alters the expression of antiapoptotic proteins. RMS-13 cells were treated with *viscum*, *TT*, and *viscumTT* in increasing concentrations for 24 h. Afterwards, cells were lysed and whole proteins lysates were analysed by Western blot for altered expression of X-linked inhibitor of apoptosis protein (XIAP), baculoviral IAP repeat containing 5 (BIRC5), myeloid cell leukemia 1 (MCL1), B-cell lymphoma 2 (BCL2), and BCL2 like 1 (BCL2L1). *β*-actin served as a loading control. Mistletoe lectin (ML) and oleanolic acid (OA) concentrations were used as marker substances for *viscum* and *TT*, respectively.

To further elucidate the underlying molecular mechanisms of *viscum*-, *TT*-, and *viscumTT*-induced apoptosis, we analysed protein expression of apoptosis-associated proteins by Western blot analysis after 24 h treatment with mistletoe extracts. *Viscum*, *TT*, and *viscumTT* suppressed the expression of the inhibitor of apoptosis protein (IAP) family members XIAP and BIRC5. Further, incubation with *viscum* and *viscumTT* led to downregulation of BCL2 family members BCL2, BCL2L1, and MCL1 (Figure 4()).

### 3.4. *viscumTT* Inhibits Proliferation and Induces Apoptosis Ex Vivo

To verify in vitro results, we next examined the effects of *viscum*, *TT*, and *viscumTT* in primary RMS tumour cells (ex vivo). For this purpose, ex vivo cultures of primary cells of three different patients were treated with *TT*, *viscum*, and *viscumTT* in increasing concentrations for 48 h (patient no. 1) and 24 h (patient no. 2; patient no. 3), respectively.

**Figure 5:**
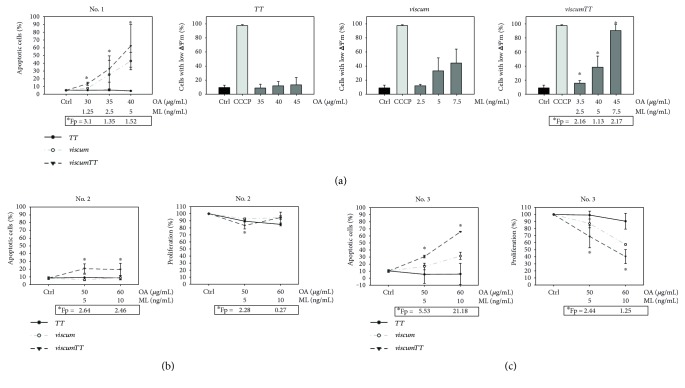
*viscumTT* induces apoptosis synergistically ex vivo. Ex vivo cultures from three different patients were incubated with *viscum*, *TT*, and *viscumTT* in increasing concentrations for 48 h (a) patient no. 1 or for 24 h, (b) patient no. 2, and (c) patient no. 3. Cell proliferation was measured by CASY cell counter analysis, and apoptosis was assessed by annexin V/PI assay and flow cytometry. All results are presented as means ± SD of two independent experiments. Webb's fractional product (^∗^Fp) was calculated to assess synergism, values ^∗^Fp > 1 display synergism. Mistletoe lectin (ML) and oleanolic acid (OA) concentrations were used as marker substances for *viscum* and *TT*, respectively.

Patient no. 1 was incubated with lower concentrations of *viscum*, *TT*, and *viscumTT* than patient no. 2 and patient no. 3, but we changed the time course and extended the incubation time to 48 h. *Viscum* and to a more pronounced extent *viscumTT* effectively induced apoptosis and decreased ∆*Ψ*_m_, while *TT* showed no cytotoxic effect ex vivo (Figure 5(a)). Patient no. 2 showed only a moderate increase of apoptotic cells after treatment with *viscumTT*, while neither *TT* nor *viscum* inhibited cell proliferation or induced apoptosis effectively after 24 h incubation (Figure 5(b)). After 24 h incubation with *viscum* and *viscumTT*, we were able to detect a concentration-dependent inhibition of proliferation as well as induction of apoptosis in patient no. 3 (Figure 5(c)).

### 3.5. *viscumTT* Effectively Inhibits Tumour Growth In Vivo

**Figure 6:**
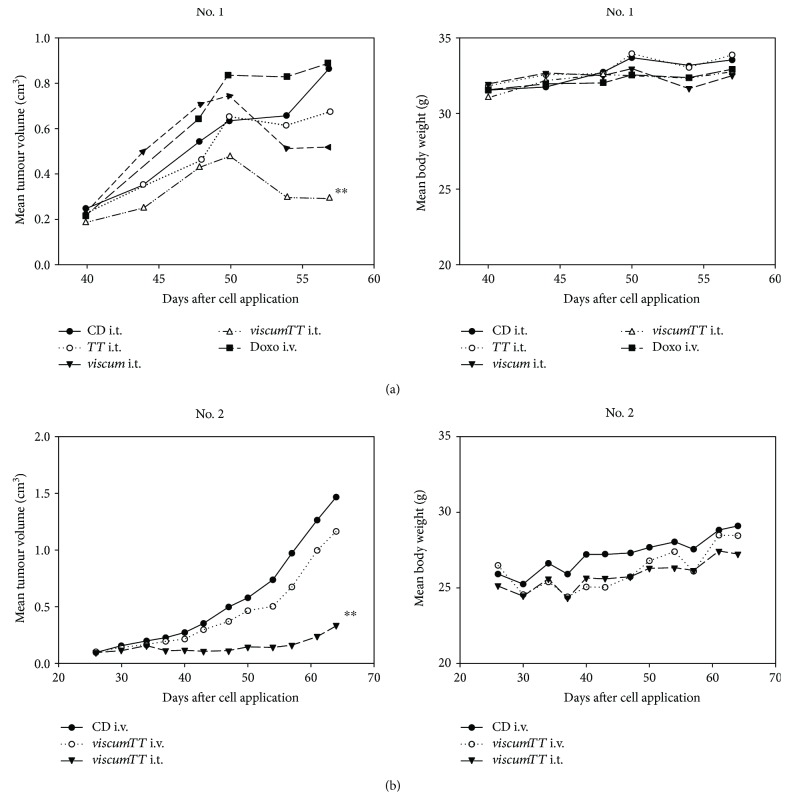
*viscumTT* effectively reduces tumour volume in patient-derived RMS xenografts. Patient-derived RMS cells from (a) patient no. 1 and (b) patient no. 2 were used for s.c. implantation in the inguinal region of mice. Treatment started on day 12 when tumours were palpable, with (a) i.t. administration of *viscum*, *TT*, *viscumTT*, cyclodextrins (CD; control group), and i.v. doxorubicin (Doxo) and (b) i.t. administration of *viscumTT* and i.v. treatment with *viscumTT* and cyclodextrins. The mice were treated every two-three days in rising concentrations, and each dose was given twice. The administered concentrations were 40/60/80 mg/kg oleanolic acid (*TT*), 0.5/1.0/1.5 *μ*g/kg mistletoe lectin (*viscum*), or a combination thereof (*viscumTT*). Two-way ANOVA and Bonferroni post hoc tests were applied to determine differences between mouse xenograft treatment groups (^∗∗^*p* ≤ 0.01).

To validate our in vitro and ex vivo findings, we established two patient-derived xenografts (PDX) to examine the effects of *viscum*, *TT*, and *viscumTT* in vivo. For this purpose, we focused on two different study designs. In PDX no. 1, we examined the therapeutic effect of i.t.-administered *viscumTT* compared to *viscum*, *TT*, and doxorubicin. In PDX no. 2, however, we investigated the therapeutic effectiveness of i.t. and i.v. application. Therefore, NMRI-nu/nu mice were treated with *viscum*, *TT*, and *viscumTT* (i.v. and/or i.t.). Control groups received the vehicle cyclodextrin i.v. or i.t. Extracts were administered every two to three days in increasing concentrations, and each concentration was given twice. During treatment, the mice showed no significant loss of body weight, and the administered extracts were well tolerated. The mean tumour volumes were calculated (Figure 6()). In PDX no. 1, i.t. administration of *viscum* and *TT* reduced tumour volume effectively and *viscumTT* led to a significant inhibition of tumour volume in comparison to the control group (Figure 6(a)), whereas doxorubicin as a standard therapeutic drug had no effect. In PDX no. 2, *viscumTT* i.t. was more effective than *viscumTT* i.v. and both application forms inhibited tumour volume significantly compared to control mice (Figure 6(b)).

## 4. Discussion

This study shows that *viscum* and *viscumTT* exerted strong antiproliferative and apoptosis-inducing effects on RMS in vitro and ex vivo, whereas *TT* showed only moderate inhibitory effects. RH-30 cells were more resistant to *viscum* treatment than RMS-13 cells. It is known that cytotoxic effects of ML, the main component of the *viscum* extract, differ in cell lines, probably due to differences in expression of ML binding sites on the cell surface [49, 50]. The marker substance of the *TT* extract OA, which exert cytotoxic effects in various cancer cell lines [37, 46, 51], did not induce apoptosis in either RMS cell line effectively, which might be due to low OA concentrations. The whole mistletoe extract, *viscumTT*, proved to be more potent than the single extracts and displayed a synergistic effect on apoptosis induction in RMS cells in vitro and ex vivo. This result is in line with the assumption that whole plant extracts can be more effective than their single constituents [52–54]. Synergistic effects of *viscumTT* were already recently shown in other paediatric tumour cell lines [41–44].

**Figure 7:**
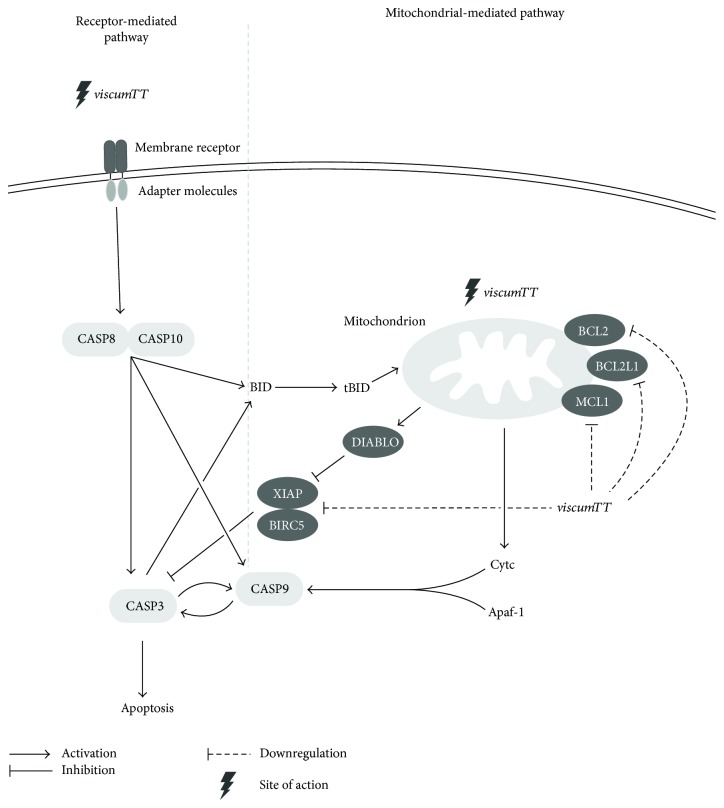
Scheme for *viscumTT*-induced apoptosis via the extrinsic and the intrinsic signalling pathways. Through yet unknown mechanisms, *viscumTT* activates caspase-8 (CASP8) and caspase-10 (CASP10), resulting in an activation of the caspase cascade. Caspase-9 (CASP9) acts as an effector downstream of CASP8 and CASP10 rather than an initiator of apoptosis. Further, *viscumTT* downregulates antiapoptotic proteins B-cell lymphoma 2 (BCL2), BCL2 like 1 (BCL2L1), and myeloid cell leukemia 1 (MCL1) as well as inhibitors of apoptosis proteins baculoviral IAP repeat containing 5 (BIRC5) and X-linked inhibitor of apoptosis protein (XIAP), thus shifting the balance towards apoptosis.

*Viscum* and *viscumTT* triggered apoptosis in RMS via the extrinsic as well as the intrinsic apoptosis signalling pathway, evidenced by mitochondrial membrane depolarisation and activation of CASP8 and CASP9 (Figure 7). This is in line with results from several studies, which reported that ML as well as OA induced apoptosis by affecting different apoptosis pathways. Aqueous mistletoe extracts as well as purified ML proved to exert cytotoxic effects in various cancer cell lines either by the intrinsic [21, 25, 55] or additionally by the extrinsic [41, 56] apoptosis signalling pathway. OA and its derivatives are also known to invoke both the extrinsic [57–62] as well as the intrinsic [37–39] signalling pathway. Furthermore, BA could induce apoptosis via the mitochondrial signalling pathway in RMS [63] and neuroblastoma [64]. Additionally, BA and OA have been shown to interact directly with mitochondrial membranes [64–66].

Caspase inhibitory assays showed that *viscum*- and *viscumTT*-induced apoptosis was caspase-dependent. Interestingly, caspase inhibitors were more effective in preventing apoptosis in cells treated with *viscum* rather than *viscumTT*. Addition of triterpene acids to aqueous mistletoe extracts seems to activate as yet unknown mechanisms, which enhance apoptosis induction caspase independently. Caspase-independent apoptosis induction by OA was also observed by Konopleva et al. [66].

Inhibition of the extrinsic pathway by CASP8 and CASP10 inhibitors reduced apoptosis significantly. Notably, the CASP10 inhibitor was more effective in reducing apoptosis than the pan-caspase inhibitor, underlining the dependency of *viscum*- and *viscumTT*-mediated apoptosis induction on CASP10. CASP10 is a homologue of CASP8 and activated through the receptor-mediated pathway. However, some studies indicate involvement of CASP10 in apoptosis induction by cytotoxic agents in a FADD-dependent but receptor-independent manner [67, 68].

Interestingly, the CASP9 inhibitor was not only ineffective in *viscumTT*-treated cells, but enhanced *viscumTT*-induced cell death. This result is in line with the previous results, which reported augmented apoptosis after treatment with inhibitors of CASP9 in combination with oridonin [69]. Also, CASP9 inhibition is suspected to block autophagic flux and thus to promote caspase-independent cell death [69, 70]. However, caspase inhibitor assays suggest that CASP9 does not act as an initiator but merely as an effector caspase in an amplification loop and is activated by CASP8 or CASP10 similar to TNF-*α*, which induced apoptosis via activation of CASP9 by CASP8 [71, 72]. Additionally, the mitochondrial and receptor-mediated pathways are crosslinked by the cleavage of BH3 interacting domain death agonist (BID) into truncated BID (tBID) by CASP8 or CASP10. Also, cotreatment with the OA derivative C-28 methyl ester of 2-cyano-3,12-dioxoolean-1,9-dien-28-oic acid (CDDO-Me) and CASP8 inhibitor CASP9 activity was decreased, suggesting that CASP9 activation is downstream of CASP8 [73].

To illuminate the underlying mechanisms of *viscum-*, *TT-*, and *viscumTT*-induced apoptosis further, apoptosis-associated proteins were analysed by Western blot analyses to detect altered expression. The results showed that *viscum* and even more prominently *viscumTT* shifted the balance towards apoptosis by changing the ratio of pro- and antiapoptotic proteins.

After treatment with *viscum* and *viscumTT*, downregulation of the antiapoptotic BCL2 family members BCL2 and BCL2L1 was detected. Overexpression of BCL2 family members is often found in cancers and contributes to apoptosis resistance. Genetic mutations, for example, gli-amplification and a positive PAX3/FOXO1 fusion status, and increased activity of transcription factor signal transducer and activator of transcription 3 (STAT3) lead to overexpression of BCL2 and BCL2L1 in RMS [74]. Downregulation of BCL2 and BCL2L1 was found after treatment with *viscum* [42] and OA [75, 76]. MCL1, another antiapoptotic BCL2 family member, which is upregulated in RMS [77], is also downregulated by *viscum* and *viscumTT*. This is in line with the previous results of decreased expression of MCL1 after treatment with ML [21, 78] and *viscumTT* [43]. Furthermore, Ryu et al. have shown inhibition of the STAT3 pathway with downregulation of BCL2L1, BIRC5, and MCL1 after incubation with CDDO-Me [79]. Moreover, *viscum* and *viscumTT* effectively reduced expression of IAP family members BIRC5 and XIAP. IAP family members prevent apoptosis by directly inhibiting caspases or the assembly of proapoptotic complexes. BIRC5 is often highly expressed in malignant cells but not in differentiated tissues [80], and high expression levels are associated with poor prognosis [81] thus appointing it an attractive target in tumour therapy. RMS expresses high levels of BIRC5, and Caldas et al. were able to effectively reduce tumour growth by blocking BIRC5 in vivo [82]. OA treatment was able to effectively reduce BIRC5 expression in lung cancer [83], ovarian cell carcinoma [75, 84], and leukaemia [76] as well as XIAP expression in hepatocellular carcinoma [85]. Furthermore, we were able to show that *viscum*, *TT*, and *viscumTT* led to downregulation of BIRC5 and XIAP in AML [41], Ewing-sarcoma [43], and osteosarcoma [44].

Treatment with *viscumTT* seems to be more effective compared to the single compounds ex vivo and in vivo. Furthermore, *viscumTT* was more effective in reducing tumour volume than i.v. doxorubicin treatment in PDX no. 1. In addition, comparison of i.v. and i.t. treatment (PDX no. 2) showed that i.t. treatment is more effective. On the other hand, *viscumTT* can be administered in higher concentrations i.v. than i.t. Notably, *viscumTT* was more effective in vivo than in vitro. While *viscumTT* showed only moderate apoptosis-inducing properties ex vivo, tumour volume was effectively reduced in the xenograft derived from the same patient. Since mistletoe extracts are metabolised by mice, these in vitro results cannot be directly extrapolated to in vivo settings. However, *viscumTT* shows promising antitumour activity in ARMS.

## 5. Conclusions

The results show the high potential of *viscumTT* to induce apoptosis in ARMS without limiting toxicity. *viscumTT* is more effective than the single compounds and leads to a synergistic effect on apoptosis induction in vitro as well as ex vivo. In vivo *viscumTT* treatment resulted in an effective reduction of tumour volume compared to controls. In conclusion, addition of triterpene acids to aqueous mistletoe extracts enhances their cytotoxic effects in ARMS through an as yet unknown mechanism. However, *viscumTT* is a promising novel treatment approach for ARMS. Furthermore, ML exerts immunomodulating effects [86, 87]. The antiproliferative and apoptosis-inducing properties of *viscumTT* combined with the immunomodulating actions of ML provide an attractive research target not only for cancer therapy, but also for other research purposes such as rheumatic and cardiovascular diseases.

## Figures and Tables

**Table 1:** Composition of mistletoe extracts.

Extract	CD [*μ*g/mL]	ML [*μ*g/mL]	VT [ng/mL]	BA [*μ*g/mL]	OA [*μ*g/mL]
*viscum*	—	1	221.3	—	—
*TT*	230	—	—	172	4000
*viscumTT*	230	1	221.3	172	4000
